# WZB117 enhanced the anti-tumor effect of apatinib against melanoma via blocking STAT3/PKM2 axis

**DOI:** 10.3389/fphar.2022.976117

**Published:** 2022-09-16

**Authors:** Ren-Shu Zhang, Zhi-Ke Li, Jie Liu, Yao-Tiao Deng, Yu Jiang

**Affiliations:** ^1^ Department of Medical Oncology, Cancer Center, West China Hospital, Sichuan University, Chengdu, China; ^2^ Department of Oncology, The First Affiliated Hospital of North Sichuan Medical College, Nanchong, China; ^3^ School of Medical Imaging, North Sichuan Medical College, Nanchong, China

**Keywords:** apatinib, WZB117, melanoma, glycolysis, stat3, PKM2

## Abstract

**Background:** Melanoma is the most lethal skin malignant tumor with a short survival once stepping into the metastatic status and poses a therapeutic challenge. Apatinib (a tyrosine kinase inhibitor) is a promising antiangiogenic agent for the treatment of metastatic melanoma. However, antiangiogenic monotherapy is prone to acquired drug resistance and has a limited therapeutic effect. The persistence dependence of glycolytic metabolism in antiangiogenic therapy-resistant cells provides evidence that glycolysis inhibitors may enhance the effect of antiangiogenic therapy. So, this study aimed to investigate whether WZB117 (a specific GLUT1 inhibitor) could enhance the anti-tumor effect of apatinib against melanoma and its potential mechanisms.

**Methods:** We investigated the anti-tumor effects of apatinib alone or in combination with WZB117 on human melanoma cell lines (A375 and SK-MEL-28). The MTT assay determined cell viability and the half-maximal inhibitory concentration (IC50). Multiple drug effect/combination indexes (CI) analysis was conducted to assess interactions between apatinib and WZB117. Signal transducer and activator of transcription 3 (STAT3) pathway measured by western blotting and immunofluorescence staining. RNA expression analyses were performed using the reverse transcription-quantitative PCR method.

**Results:** Apatinib and WZB117 showed dose and time-dependent growth inhibitory effects in both melanoma cells. The IC50 of apatinib at 48 h in A375 and SK-MEL-28 cells was 62.58 and 59.61 μM, respectively, while the IC50 of WZB117 was 116.85 and 113.91 μM, respectively. The CI values of the two drugs were 0.538 and 0.544, respectively, indicating a synergistic effect of apatinib combined with WZB117. We also found that glucose consumption and lactate production were suppressed by apatinib plus WZB117 in a dose-dependent manner, paralleled by reducing glycolytic enzyme pyruvate kinase M2 (PKM2). The potential mechanism of the combination was to suppress the phosphorylation of STAT3. Knockdown of STAT3 by siRNA inhibited the expression of PKM2, while the activation of STAT3 by IL-6 increased the expression of PKM2. The effects of IL-6 were attenuated by apatinib combined with WZB117 treatment.

**Conclusion:** WZB117 enhanced the anti-tumor effect of apatinib against melanoma via modulating glycolysis by blocking the STAT3/PKM2 axis, which suggested the combination of apatinib with WZB117 could be a potential therapeutic candidate for melanoma.

## Introduction

Melanoma is a highly malignant tumor derived from melanocytes (Shain and Bastian, 2016). The standard-of-care treatment for localized melanoma is surgical resection with a good long-term prognosis (Gershenwald et al., 2017). However, melanoma tends to metastasize as an aggressive tumor, leading to a poor prognosis (Davis et al., 2019). For a long time the primary treatment for metastatic melanoma was dacarbazine-based chemotherapy and, in selected patients, the cytokine IL-2 therapy, with a median overall survival of fewer than 9 months (Middleton et al., 2000; Avril et al., 2004; Petrella et al., 2007). Since 2011, the treatment of metastatic melanoma has improved with targeted therapies such as BRAF and MEK inhibitors, as well as immunotherapies such as cytotoxic T lymphocyte-associated antigen four and programmed cell death protein one blocking antibodies (Luke et al., 2017). Although these novel therapies have promising efficacy for melanoma, resistance often develops over time, and many patients eventually occur disease progression and relapse (Welsh et al., 2016; O'Donnell et al., 2017). Thus, new treatment strategies are needed.

Melanoma is a highly angiogenic tumor that releases vascular endothelial growth factor (VEGF) to promote angiogenesis and carcinogenesis (Streit and Detmar, 2003). Elevated serum angiogenic factor VEGF is associated with poor prognosis in melanoma patients (Ugurel et al., 2001; Mehnert et al., 2010). Anti-vascular drugs such as endostar and bevacizumab are effective in melanoma (Cui et al., 2013; Kottschade et al., 2013). Apatinib mesylate (YN968D1) is a small molecule anti-angiogenic tyrosine kinase inhibitor. The primary mechanism of apatinib is to interfere with the binding of ATP with vascular endothelial growth factor receptor-2 and block the signal transduction after the VEGF combination, thus inhibiting tumor angiogenesis (Tian et al., 2011). Apatinib was the first anti-angiogenic drug approved by cFDA to treat metastatic gastric cancer and has been proven to be safe and effective in multiple solid tumors (Li et al., 2016; Lan et al., 2018; Zhao et al., 2019). Preclinical studies have reported that apatinib inhibits tumor growth and vasculogenic mimicry in melanoma (Liu et al., 2018). However, apatinib alone has limited efficacy, and the combination of apatinib with chemotherapy or immunotherapy may improve its effectiveness (Si et al., 2022).

Unlike normal cells, most malignant tumor cells up-regulated aerobic glycolysis and produced large amounts of lactate and macromolecular precursors regardless of their oxygen status, a phenomenon known as the “Warburg effect” (Heiden et al., 2009). Previous evidence has established that glycolysis regulates tumorigenesis and therapeutic sensitivity in melanoma, such as glycerol 3-phosphate dehydrogenase, which regulates tumorigenesis of melanoma cells by facilitating glycolysis (Ding et al., 2021). Besides, transcription factor 4 was shown to sensitize melanoma cells to vemurafenib by inhibiting glucose transporter 3-mediated glycolysis. Glucose transporter 1 (GLUT 1), a rate-limiting factor of glucose transport in tumor cells, is frequently upregulated in multiple solid tumors and associated with more aggressive phenotypes and poor survival (Fu et al., 2016; Wang et al., 2017). WZB117 is a synthetic small molecule GLUT1 inhibitor, which has been shown to have anticancer activity against multiple solid tumors, providing a promising option for further development of anticancer therapies (Liu et al., 2012; Li et al., 2019; Shima et al., 2022). However, the antitumor effects of WZB117 on melanoma remain unclear.

In our current work, we sought to explore the effects of apatinib in combination with WZB117 functioned in melanoma cells. Moreover, we gain further insight into the mechanisms of apatinib combined with WZB117 on melanoma from the perspective of tumor metabolism.

## Materials and methods

### Reagents and antibodies

Dimethyl sulfoxide (DMSO), 3-(4,5-dimethylthiazol-2-yl)-2,5 diphenyltetrazolium bromide (MTT), and apatinib were purchased from MCE (NJ 08852, United States); WZB117 and S3I-201 were purchased from Selleckchem (Houston, TX, United States); Annexin V-FITC/PI apoptosis detection kit was purchased from KeyGen Biotech (Nanjing, China); recombinant human IL-6 was obtained from PeproTech (Rocky Hill, United States). Fetal bovine serum (FBS) and Dulbecco’s modified eagle medium (DMEM) were purchased from Gibco (Brooklyn, NY). All the other materials used were also of analytic reagent grade and were used without further purification. Antibodies against STAT3, hexokinase 2 (HK2), pyruvate kinase isozyme 2 (PKM2), PFKFB3, and hypoxia-inducible factor-1α (HIF-1α), goat anti-rabbit and anti-mouse immunoglobulin G (IgG) secondary antibodies, as well as goat anti-rabbit IgG H&L (Alexa Fluor 488), were obtained from Zen bioscience (Chengdu, China). Antibodies against phosphorylated (p)-STAT3 were purchased from Cell Signaling Technology (Danvers, MA, United States). The p-VEGFR2 antibody was from Affinity Biosciences (OH, United States).

### Cell culture

Human melanoma cell lines A375 and SK-MEL-28 were obtained from American Type Culture Collection (ATCC, United States). Both cell lines are cultured in DMEM complete medium containing 10% FBS, with the addition of 100 μg/ml streptomycin and 100 U/mL penicillin, at 37°C with 5% CO_2_ in a humidified atmosphere.

### *In vitro* cell proliferation assays

The half maximal inhibitory concentration (IC50) of apatinib and WZB117 in A375 and SK-MEL-28 melanoma cells were determined using an MTT assay. Briefly, both cells were harvested and seeded in 96 well plates (1,000–3,000 cells per well). After incubation overnight, the cells were exposed to various concentrations of drugs (apatinib: range from 0 to 100 μM; WZB117: range from 0 to 160 μM) for 24 h/48 h/72 h. Following apatinib or WZB117 treatment, 20 μl of 0.5 mg/ml MTT was added to each well and incubated for another 4 h at 37°C. Then the supernatants were carefully removed and added 150 μl DMSO per well. At last, the light absorbance at 570 nm was determined in a luminescence plate reader (PerkinElmer, United States) according to the manufacturer’s instructions. Furthermore, IC50 values of apatinib and WZB117 were calculated using GraphPad Prism 8.0.2 (GraphPad Software, Inc., La Jolla, CA, United States). At least three replicates were performed for each assay.

### The combination index of apatinib plus WZB117

According to Chou and Talalay’s median effect principle, the synergistic effect of apatinib and WZB117 was determined by the combination index (CI) method (Chou, 2007). In brief, we fixed WZB117 at a certain concentration and combined it with different concentrations of apatinib, and cell proliferation assays were conducted by MTT assay and the CompuSyn™ program (Biosoft, Ferguson, MO) was used for analysis. The equation used to calculate the dose-effect profiles of combined apatinib with WZB117 was CI = DA/DA1 + DB/DB1, where DA and DB are the concentrations of drug A (apatinib) drug B (WZB117), respectively. DA1 and DB1 represent the dose of single drugs that generate the same effect. CI values < 1 indicate synergism, CI = 1 indicates an additive effect, and CI > 1 corresponds to an antagonism effect.

### Colony formation assay

A375 and SK-MEL-28 melanoma cells were plated on a 6 well plate (600 cells per well) and were treated in the following four groups: control group, apatinib group, GLUT1 inhibitor- WZB117 group, apatinib and WZB117 combination group. The concentration of apatinib was 20 μM, and WZB117 was 22.5 μM, according to the results of previous experiments. After 48 h, the drugs were removed, and the cells were incubated with fresh complete DMEM until there were visible colonies. Cells were washed with sterile phosphate buffer (PBS), fixed with methanol for 10 min, and then stained with 0.1% crystal violet for 20 min. The clones were photographed and counted in five random files under the microscope. All experiments were performed in triplicate.

### Wound healing assay

Lateral cell migration ability was assessed by wound healing assay. A375 and SK-MEL-28 cells were seeded into 6 well plates, respectively. After incubation overnight, cell monolayers in each well were scratched with a 200 μl pipette tip in a straight line to create a scratch. Then the cells were washed with cold sterile PBS thrice and then replaced with 2 ml DMEM containing different drugs for 48 h. The concentration of apatinib was 20 μM, and WZB117 was 22.5 μM, respectively. It is very important to create scratches of similar width in the four groups. The scratch lines were photographed at 0 and 48 h using an inverted phase-contrast microscope equipped with a digital camera (Olympus, Tokyo, Japan). By comparing the scratch field at 0 and 48 h (quantify by ImageJ), the changed field of the four groups was obtained and calculated as cell motility area = changed area/area at 0 h, changed area = area at 0 h-area at 48 h. Each experiment was performed in triplicate.

### Transwell assay

A transwell assay determined vertical cell migration ability. A375 and SK-MEL-28 cells (1 × 10^5^ cells per chamber) were seeded in the transwell upper chambers (24 wells, 8 μm pore size, Corning Costar, Cambridge, MA) with complete DMEM medium and incubated overnight. Then the cells were washed and treated with different drugs (apatinib was 20 μM and WZB117 was 22.5 μM, respectively) in 200 μl serum-free DMEM medium for 48 h, and 600 μl DMEM medium containing 15% FBS was added to the lower chamber at the same time. The cells in the upper chamber were removed carefully, while cells in the bottom chambers were fixed in methanol for 10 min and stained with 0.1% crystal violet for 20 min at room temperature and were photographed and recorded images of the migrated cells.

Cell invasion ability was also performed by transwell assay similar to the vertical cell migration study with some different steps. In brief, cold upper inserts were coated with 60 μL matrigel (Corning, United States) per chamber and then placed at 37°C for gelation. The dilution agent was DMEM without FBS. 2 × 10^5^ cells per well suspended in 200 μl FBS free DMEM medium were added into the upper chamber, while 600 μl DMEM medium containing 15% FBS was placed in the lower chamber. The others followed the steps of the transwell migration assay. At last, the cells were counted in 5 random visual fields for each chamber using an inverted phase-contrast microscope (Olympus, Japan).

### Apoptosis assay

Annexin V-FITC and propidium iodide (PI) staining assay was used to determine melanoma cell apoptosis by an apoptosis detecting kit (KeyGen, Nanjing, China) according to the manufacturer’s instructions. In brief, A375 and SK-MEL-28 cells were plated in 24 well plates in cultured standard growth conditions overnight, then exposed to various drug treatments for 48 h. The concentration of apatinib was 20 μM, and WZB117 was 22.5 μM, respectively. After being harvested by trypsin solution, cells were washed with cold PBS thrice and then centrifuged at 1,500 rpm/min for 5 min. The cells were resuspended in a binding buffer of 100 μl and incubated with 5 μl Annexin V-FITC on ice in the dark condition for 15 min, followed by the addition of 5 μl PI solution to each group. After that, the percentage of cells in the early and late stages of apoptosis was determined quickly by flow cytometry within 30 min.

### Analysis of glucose consumption and lactate production

A375 and SK-MEL-28 cells were seeded in six well plates (1–1.5 × 10^5^ cells per well) for lactate production measurement and were seeded in 12 well plates (3–5 × 10^4^ cells per well) for glucose consumption measurement. After different treatments for 48 h, the culture medium was collected and centrifuged to remove the cell debris. The glucose and lactate concentrations were measured using the glucose oxidase method (Nanjing Jiancheng Biotechnology, Nanjing, China) and with a lactic acid assay kit (Solarbio, Beijing, China) according to the manufacturer’s instructions. Differences in glucose consumption and lactate production were determined by the relative proportions compared to the control group. The concentrations of lactate or glucose were measured and normalized by cell numbers.

### Gene silencing by siRNA

A375 and SK-ML-28 cells were seeded into 12-well plates (3–5 × 10^4^ cells per well) or 6-well plates (1–1.5 × 10^5^ cells per well), and small interfering RNA (siRNA) was transfected with Lipofectamine 8,000 reagent (Beyotime). Three STAT3-specific siRNAs (si-STAT3-1, si-STAT3-2, and si-STAT3-3) were used to knock down STAT3, and non-silencing siRNAs (si-NC) were used as a negative control (Tsingke, Beijing, China). siRNAs were transfected at the final RNA concentration of 50 nM and cultured for 48 h/72 h for further experiments. The human siRNA sequence transfected in this study is as follows:

STAT3-1: sense, 5′-CAC​AAU​CUA​CGA​AGA​AUC​ATT-3′;

Antisense, 5′-UGA​UUC​UUC​GUA​GAU​UGU​GTT-3′.

STAT3-2: sense, 5′-GUC​AUU​AGC​AGA​AUC​UCA​ATT-3′;

Antisense, 5′-UUG​AGA​UUC​UGC​UAA​UGA​CTT-3′.

STAT3-3: sense, 5′-CCA​ACA​AUC​CCA​AGA​AUG​UTT-3′;

Antisense, 5′-ACA​UUC​UUG​GGA​UUG​UUG​GTT-3′.

### Quantitative real-time polymerase chain reaction assay

Total RNAs of A375 and SK-MEL-28 cells were isolated using the TRIzol reagent (Takara Bio, Inc., Otsu, Japan). The cDNA synthesis was performed using a PrimeScript™ RT reagent Kit (Perfect Real Time; Takara Biotechnology, Shiga, Japan). Real-time PCR was performed in a LightCycler^®^ 96 system (Roche, Switzerland) with TB Green™ Premix Ex Taq™ II (Tli RNaseH Plus) (Takara Biotechnology, Shiga, Japan) according to the manufacturer’s protocol. β-actin was used as internal control, and relative expressions were analyzed using the 2^−ΔΔCT^ relative quantification method. The primers used for real-time PCR detection in this study were as listed:

β-actin, forward:5′-CATGTACGTTGCTATCCAGGC-3′;

Reverse:5′-CTCCTTAATGTCACGCACGAT-3′.

STAT3, forward:5′-GGGAGAGAGTTACAGGTTGGACAT-3′;

Reverse:5′-AGACGCCATTACAAGTGCCA-3′.

PKM2, forward:5′-ATTATTTGAGGAACTCCGCCG-3′;

Reverse:5′-ATTCCGGGTCACAGCAATGAT-3′.

### Western blot analysis

After different treatments at indicated time points, cells were harvested and lysed using RIPA protein extraction reagent (Beyotime) with a protease inhibitor cocktail. Protein concentrations were determined using a BCA protein assay kit (Solarbio, Beijing, China). Equal amounts of proteins were electrophoresed on 6%–10% SDS-PAGE gels and then transferred to polyvinylidene difluoride (PVDF) membranes. Membranes were blocked in 5% free-fat milk in TBST buffer, incubated overnight at 4°C with the primary antibodies, and then visualized using a chemoluminescence assay (Omni-ECL, Epizyme Biotech, Shanghai, China). The expression level was normalized by the β-actin expression level of each sample.

### Immunofluorescence staining assay

A375 and SK-MEL-28 cells were spread on coverslips, incubated overnight, and then exposed to various drug treatments for 48 h. The cells were fixed, permeabilized with Triton X-100 on ice, and incubated with anti-STAT3 overnight at 4°C. The cells were incubated with Alexa Fluor^®^488 for 1 h, and the nuclei were stained with DAPI for 5 min. Finally, the coverslips were detected using a fluorescence microscope.

### Statistical analysis

All data (means ± SD) analysis was conducted by SPSS 17.0 software and Graph Pad Prism 8.0.2 (Graph Pad Software, Inc., La Jolla, CA, United States). Multiple samples were analyzed by one-way analysis of variance, and a two-sided Student’s *t* test was used for comparison of two groups. Values of *p* < 0.05 were considered statistically significant.

## Results

### Apatinib combined with WZB117 showed synergistic cell growth inhibitory effects on A375 and SK-MEL-28 cells

**FIGURE 1:**
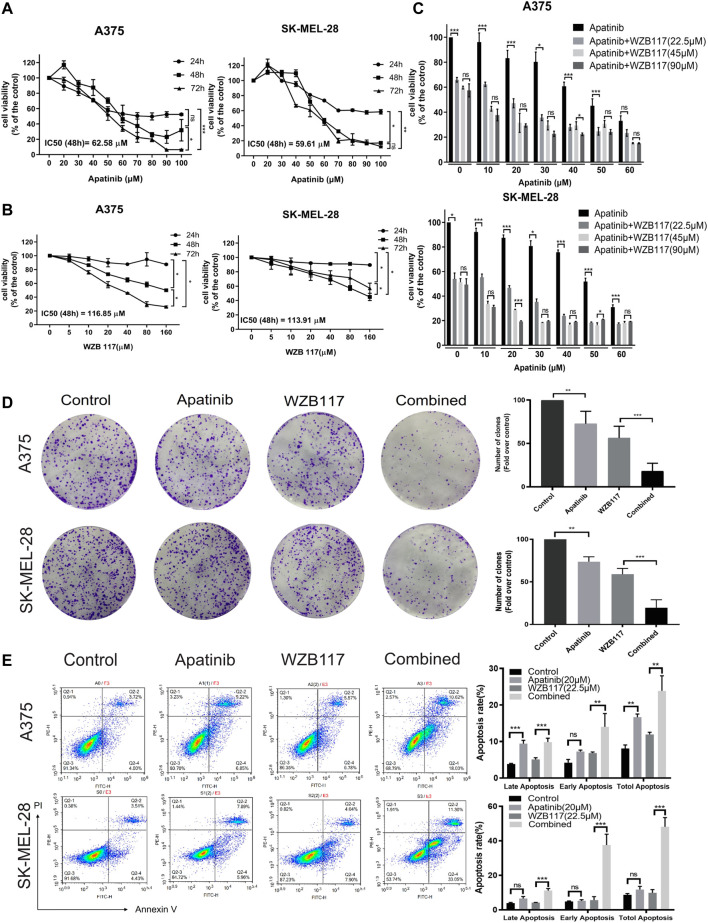
Apatinib combined with WZB117 showed synergistic cell proliferation inhibitory effects in A375 and SK-MEL-28 cells. **(A)** Dose-response curves of both cells when treated with a range of concentrations of apatinib. **(B)** Dose-response curves of both cells was treated with a range concentration of WZB117. **(C)** Cell viability of both cells when treated with a range concentration of apatinib combined with different concentrations of WZB117 for 48 h. **(D)** Apatinib and WZB117 inhibit colony formation. The images of colony formation are displayed in the *left* panels, and quantitative results are displayed in the *right* panels. Quantitative data are presented as the mean ± SD of five independent experiments. **(E)** Apatinib and WZB117 induce cell apoptosis. The representative dot plots are shown in the *left* panels, and quantitative results are shown in the *right* panels. Quantitative data are presented as the mean ± SD of three independent experiments. “*” means *p* <0.05, “**” means *p* <0.01, and “***” means *p* <0.001.

To investigate the toxicity of apatinib and WZB117 in melanoma cells, the viability of A375 and SK-MEL-28 cells after exposure to various concentrations of apatinib and WZB117 for 24 h/48 h/72 h were determined by MTT assays. The results showed that apatinib inhibited the proliferation of A375 and SK-MEL-28 cells in a dose and time-dependent manner (Figure 1A). However, the survival rate of A375 and SK-MEL-28 cells were maintained at over 80% after being treated with 160 μM WZB117 for 24 h (Figure 1B). At the 48-h time point, the IC50 values of apatinib were 62.58 ± 4.05 μM and 59.61 ± 4.02 μM for A375 and SK-MEL-28 cells, respectively. The IC50 of WZB117 at 48 h were 116.85 ± 44.81 μM and 113.91 ± 23.86 μM for A375 and SK-MEL-28 cells, respectively.

To investigate the cytotoxicity efficiency of combination therapy, cells were treated with dose-escalating of single apatinib and apatinib combined with WZB117 for 48 h and assessed viability via MTT assay. We selected 22.5, 45, and 90 μM as low, medium, and high levels of WZB117, combined with a range concentration of apatinib, which was 0, 10, 20, 30, 40, 50, and 60 μM based on results from preliminary tests. The combination of apatinib with WZB117 further reduced cell activity **(** Figure 1C **)**. We further calculated the CI of apatinib combined with WZB117 based on MTT results to evaluate the effect of the interaction between the two drugs. The results showed that the CI of apatinib combined with WZB117 was lower than 1 at all the concentrations tested, indicating a synergistic effect of apatinib with WZB117. Especially, we observed strong synergistic growth inhibition effects in A375 (CI = 0.538) and SK-MEL-28 cells (CI = 0.544), respectively, when at a molar ratio of 1:1.125 (apatinib 20 μM vs. WZB117 22.5 μM).

### Apatinib combined with WZB117 synergistically inhibits the proliferation, migration and invasion and induces apoptosis of melanoma cells

To further investigate the effects of combined therapy, we divided cells into four experimental groups: the control group, the apatinib group, the WZB117 group, and the apatinib plus WZB117 group (the combination group). First, we conducted a colony formation assay to explore whether the combination therapy could exert a synergistically long-term proliferation inhibitory effect on A375 and SK-MEL-28 cells. The results showed that apatinib and WZB117 monotherapy inhibited clone formation in both melanoma cells. The number of A375 and SK-MEL-28 cells colonies reached the lowest point in the combination group, much lower than that of apatinib and WZB117 alone **(** Figure 1D **)**. Apatinib and/or WZB117 on cell apoptosis were also studied. The results of Annexin V-FITC and PI staining assay showed that the apatinib or WZB117 monotherapy could induce apoptosis of A375 cells, but it was not prominent in SK-MEL-28 cells. The combined treatment synergistically promoted cell apoptosis proportion up to 28.56% for A375 cells and 44.35% for SK-MEL-28 cells, respectively, and there was a significant difference (Figure 1E **)**.

**FIGURE 2:**
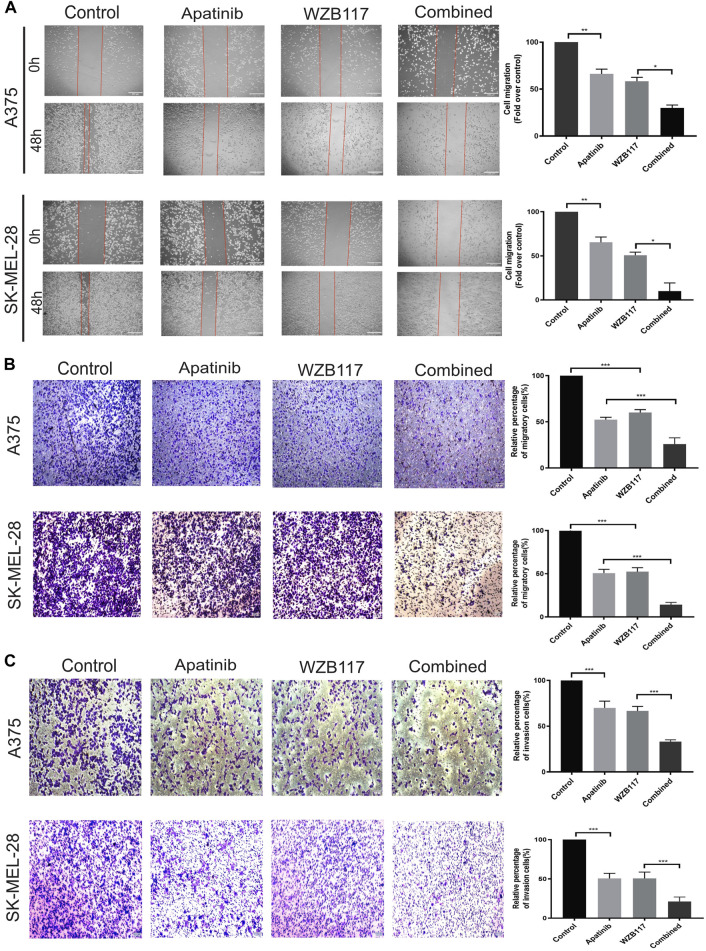
Apatinib combined with WZB117 showed synergistic migration and invasion inhibitory effects in A375 and SK-MEL-28 cells. **(A)** Apatinib and WZB117 inhibit lateral migration ability. The microscopic appearance of scratches in both cells at 0 and 48 h are shown in the *left* panels, and relative quantification data are shown in the *right* panels. Scale bar = 200 µm. Quantitative data are presented as the mean ± SD of three independent experiments. **(B)** Apatinib and WZB117 inhibit vertical migration ability. The microscopic images are shown in the *left* panels, and relative quantification data are shown in the *right* panels. Scale bar = 100 µm. Quantitative data are presented as the mean ± SD of five independent experiments. **(C)** Apatinib and WZB117 inhibit invasion ability. The microscopic images are shown in the *left* panels, and relative quantification data are shown in the *right* panels. Scale bar = 100 µm. Quantitative data are presented as the mean ± SD of five independent experiments. “*” means *p* <0.05, “**” means *p* <0.01, and “***” means *p* <0.001.

To analyze the influence of apatinib combined with WZB117 on the metastatic potency of melanoma cells, wound healing test and transwell migration assay were performed to evaluate the lateral and vertical migration ability of A375 and SK-MEL-28 cells, respectively. The results showed that the lateral migration abilities of cells in the apatinib and WZB117 monotherapy group were lower than that in the control group **(** Figure 2A **)**. Meanwhile, the transwell migration assay also showed similar inhibition of apatinib and WZB117 on the vertical migration ability of melanoma cells (Figure 2B **)**. It is noteworthy that the lateral and vertical migration inhibition effect of apatinib combined with WZB117 is significantly stronger than that of apatinib or WZB117 monotherapy **(** Figures 2A,B **)**. Consistent with these results, the transwell invasion assay showed that apatinib and WZB117 significantly suppressed the invasion capacity of the two melanoma cells, while the combination of apatinib and WZB117 synergistically reduced migrating cell number **(** Figure 2C **)**.

### Apatinib combined with WZB117 suppresses STAT3 activation

**FIGURE 3:**
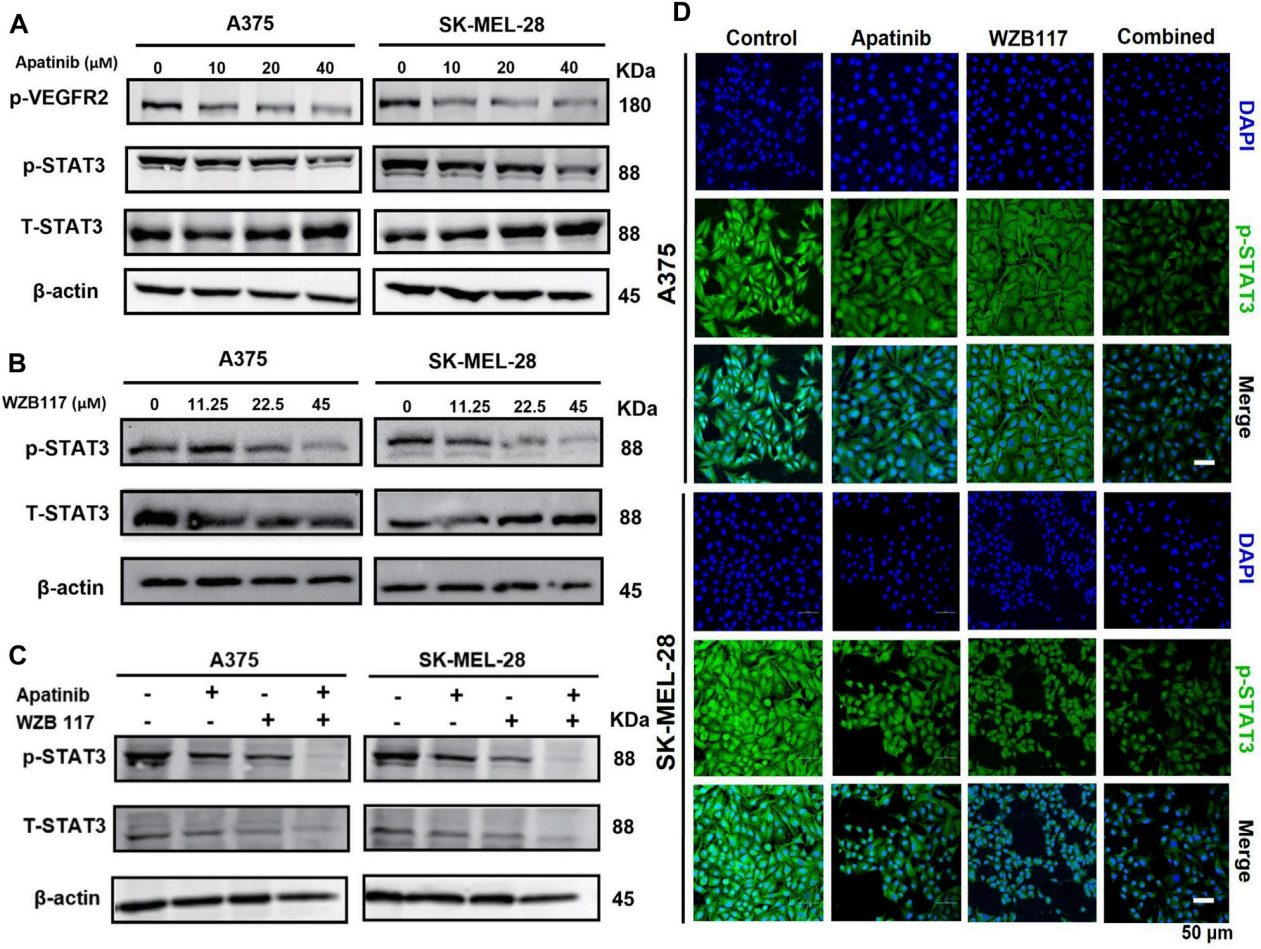
Effect of apatinib combined with WZB117 on the STAT3 pathway in A375 and SK-MEL-28 cells. **(A)** Western blot analysis of protein expressions of p-VEGFR2, STAT3, and p-STAT3. Both cells were treated with a range concentration of apatinib for 48 h. **(B)** Western blot analysis of protein expressions of STAT3 and p-STAT3. Both cells were treated with a range concentration of WZB117 for 48 h. **(C)** Western blot analysis of protein expressions of STAT3 and p-STAT3. **(D)** Immunofluorescence staining of p-STAT3. Both cells were treated with apatinib and WZB117, either alone or in combination, for 48 h (p-STAT3: green; DAPI: blue). Scar bar = 50 μm.

We found that the expression of phospho-VEGFR2 (Tyr1175) was decreased in a dose-dependent manner for A375 and SK-MEL-28 cells after being treated with different concentrations (0, 10, 20, or 40 µM) of apatinib **(** Figure 3A). We next assessed the effect of apatinib and WZB117 on phosphorylation of STAT3, a transcription factor regulated by VEGFR2. Western blot results showed that apatinib suppressed the expression of phospho-STAT3 in a dose-dependent manner **(** Figure 3A **)**. Interestingly, we also found that WZB117 inhibited STAT3 phosphorylation in melanoma cells in a dose-dependent manner (Figure 3B **)**. Our preliminary studies found that apatinib and WZB117 showed synergistic effects, so we further studied the effects of the combination of the two drugs on the STAT3 signaling pathway. Furthermore, immunofluorescence staining and western blot results showed that the combination of Aptinib and WZB117 significantly reduced phospho-STAT3 expression in A375 and SK-MEL-28 cells compared with aptinib or WZB117 alone (Figures 3C,D). These data showed that apatinib combined with WZB117 synergistically suppresses the STAT3 pathway in melanoma cells.

### STAT3 regulates glycolysis and pyruvate kinase isozyme 2 expression in melanoma cells

**FIGURE 4:**
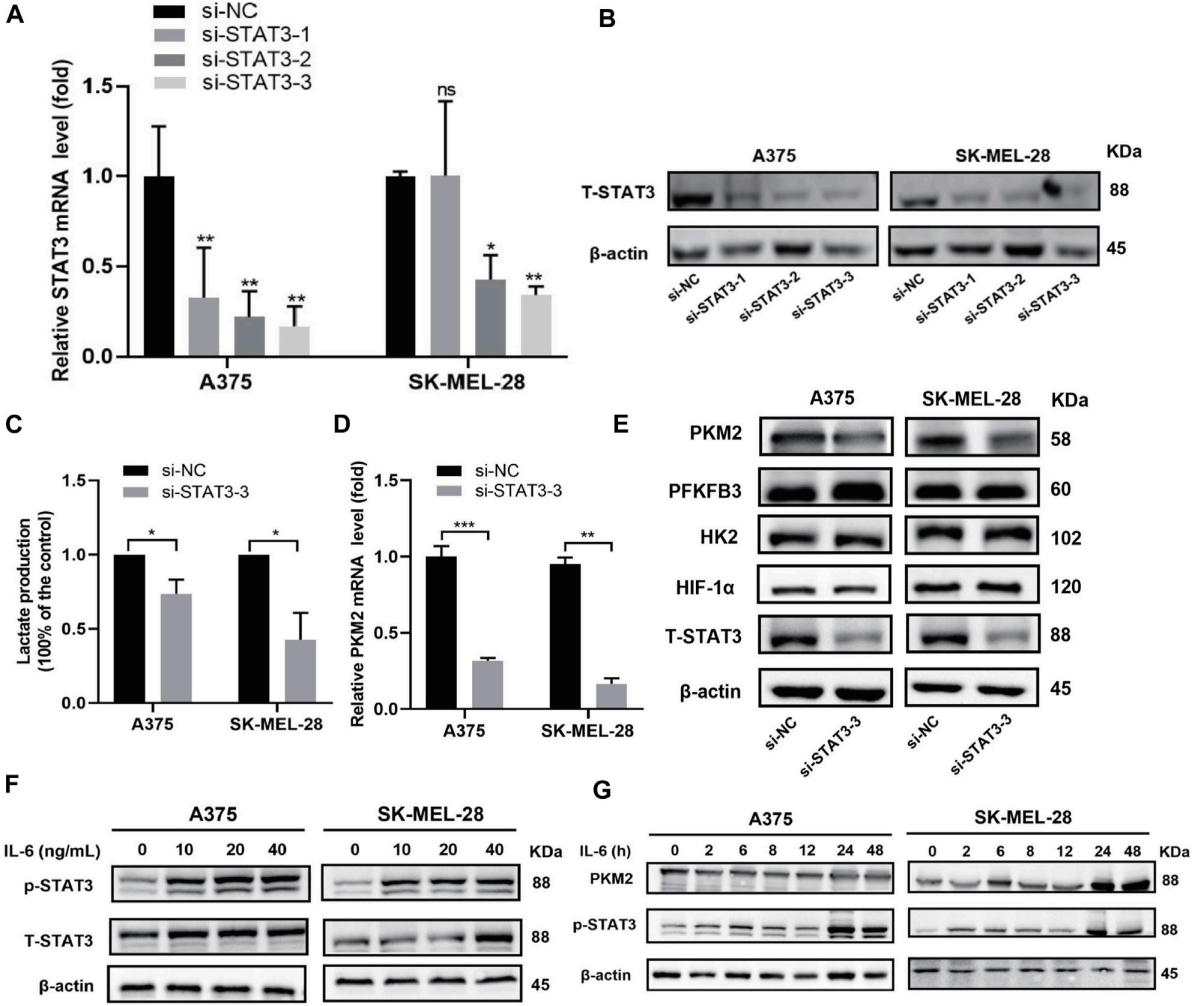
Effect of STAT3 on glycolysis and PKM2 expression in A375 and SK-MEL-28 cells. **(A)** Relative mRNA expression of STAT3 was detected by RT-qPCR assay after transfected for 48 h. Quantitative data are presented as the mean ± SD of four independent experiments. **(B)** The protein expression of STAT3 was detected by western blot assay after being transfected for 72 h. **(C)** Lactate production of both cells. Quantitative data are presented as the mean ± SD of three independent experiments. **(D)** Relative mRNA expression of PKM2 was detected by RT-qPCR assay. Data are presented as the mean ± SD of three independent experiments, except RT-qPCR assay in SK-MEL-28 cells (N = 2). **(E)** Protein expression of HK2, PFKFB3, HIF1α, and PKM2 was detected by western blot assay. **(F)** Western blot analysis of protein expressions of STAT3 and p-STAT3. Both cells were treated with a range concentration of recombinant human IL-6 for 48 h. **(G)** Western blot analysis of protein expressions of PKM2 and p-STAT3. Both cells were treated with 20 ng/ml recombinant human IL-6 for a range of duration times. “*” means *p* <0.05, “**” means *p* <0.01, “***” means *p* <0.001, and n. s., not significant (*p* > 0.05).

We investigated whether STAT3 can regulate glycolysis metabolism in melanoma cells. Firstly, we performed STAT3 knockdown experiments in A375 and SK-MEL-28 cells and tested the expression of STAT3 in different groups to verify whether the knockdown was successful. The results showed that siSTAT3-1, siSTAT3-2, and siSTAT3-3 reduce the mRNA and protein expression of STAT3 compared with the siNC group, and siSTAT3-3 showed a more obvious knockdown effect **(** Figures 4A,B **)**. So, siSTAT3-3 was selected for the following experiment. To test the role of STAT3 in glycolysis, we compared the glucose metabolism between the siNC group and the siSTAT3-3 group. As shown in Figure 4C, we noted that lactate production decreased in the siSTAT3-3 group. To further explore the mechanism of STAT3 on glucose metabolism, we evaluated the expression of key glycolytic enzymes, including hexokinase 2 (HK2), 6-phosphofructo-2-kinase/fructose-2,6-biphosphatase 3 (PFKFB3), and pyruvate kinase isozyme 2 (PKM2) and HIF-1α in A375 and SK-MEL-28 cells transfected with siSTAT3-3. Western blot results showed that the protein expression levels of HK2, PFKFB3, and HIF-1α remained following STAT3 knockdown, while the protein level of PKM2 was decreased (Figure 4E). Consistently, the mRNA expression level of PKM2 decreased significantly with STAT3 knockdown (Figure 4D **)**.

Furthermore, we used cytokines IL-6 *in vitro* to determine the effect of STAT3 activation in glycolysis. Firstly, melanoma cells were incubated with different concentrations (0, 10, 20, or 40 ng/ml) of recombinant human IL-6 for 48 h. As illustrated in Figure 4F, the expressions of p-STAT3 were elevated in both cell lines. Besides, we treated melanoma cells with 20 ng/ml IL-6 for different time points (0, 2, 6, 8, 12, 24, or 48 h). The results showed that STAT3 phosphorylation increased in A375 cells and SK-MEL-28 cells after IL-6 treatment, especially at 24 and 48 h, and the increase of PKM2 protein was consistent with the increase of STAT3 phosphorylation (Figure 4G). These results suggested that STAT3 regulates glycolytic metabolism and the expression of PKM2, a key glycolytic kinase, in melanoma cells.

### Apatinib combined with WZB117 inhibits glycolysis via STAT3

**FIGURE 5:**
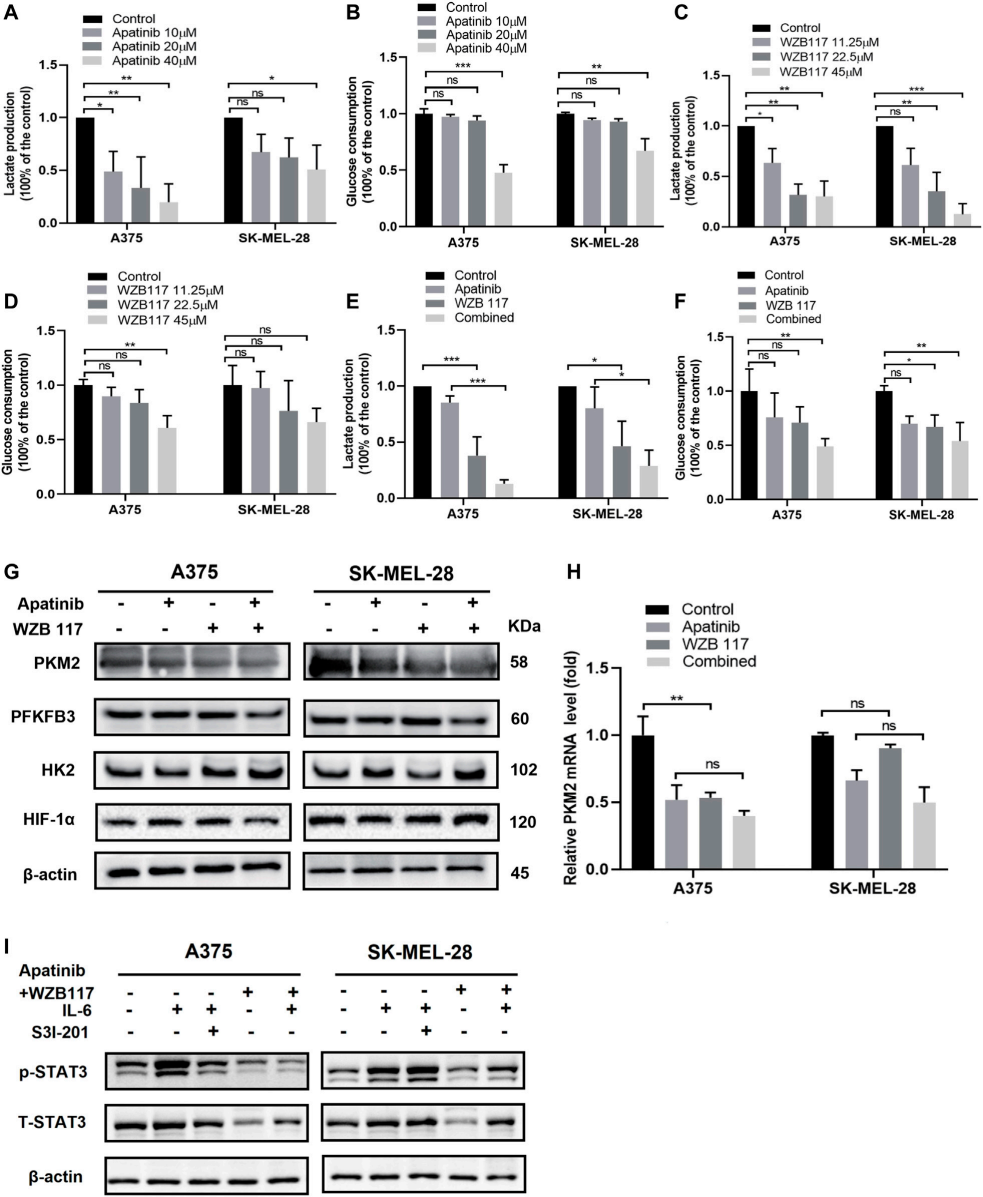
Apatinib combined with WZB117 inhibits glycolysis via STAT3 in A375 and SK-MEL-28 cells. **(A,B)** Lactate production and glucose consumption of both cells after being treated with a range concentration of apatinib for 48 h. Data are presented as the mean ± SD of three independent experiments, except for lactate production assay in A375 cells (N = 4). **(C,D)** Lactate production and glucose consumption of both cells after being treated with a range concentration of WZB117 for 48 h. Data are presented as the mean ± SD of three independent experiments, except for glucose consumption assay in SK-MEL-28 cells (N = 4). **(E,F)** Lactate production and glucose consumption of both cells after being treated with apatinib and WZB117, either alone or in combination, for 48 h. Data are presented as the mean ± SD of three independent experiments, except glucose consumption assay in A375 cells (N = 4). **(G)** Western blot analysis of protein expressions of HK2, PFKFB3, HIF1α, and PKM2. **(H)** The relative mRNA expressions of PKM2 were detected by RT-qPCR assay. Data are presented as the mean ± SD of three independent experiments. **(I)** Western blot analysis of proteins expressions of STAT3 and p-STAT3. Both cells were treated with apatinib plus WZB117 or S3I-201 for 48 h after pretreatment with recombinant human IL-6 for 1 h “*” means *p* <0.05, “**” means *p* <0.01, “***” means *p* <0.001, and n. s., not significant (*p* > 0.05).

A375 and SK-MEL-28 cells were cultured with different concentrations of apatinib (0, 10, 20, or 40 μM) and WZB117 (0, 11.25, 22.5, or 45 μM) for 48 h to detect glucose consumption and lactate production. The results revealed that the glucose consumption and lactate production of the two melanoma cells decreased in a dose-dependent manner after being treated with dose-escalating apatinib compared with the control cells **(** Figures 5A,B **)**. We also found that WZB117 significantly inhibited glucose consumption and lactate production in a dose-dependent manner in both cell lines compared to the corresponding control group (Figures 5C,D). In addition, compared with apatinib or WZB117 alone, apatinib combined with WZB117 further repressed lactate production and was accompanied by a significant reduction in glucose consumption, suggesting that apatinib combined with WZB117 may have a synergistic inhibitory effect on glycolysis in melanoma cells **(** Figures 5E,F **)**.

In order to further study the molecular mechanisms of apatinib combined with WZB117 inhibiting glycolysis level of melanoma cells, we detected the expression of key glycolysis kinases HK2, PFKFB3, PKM2, and regulatory factor HIF-1α in A375 cells and SK-MEL-28 cells treated with apatinib or WZB117 alone or in combination for 48 h. As shown in Figure 5G, the protein levels of HK2, PFKFB3, and HIF-1α changed slightly, while the expression of PKM2 was downregulated by apatinib and WZB117 monotherapy compared with the corresponding control group and further decreased in the combination group. We also detected the expression changes of PKM2 at the mRNA level and found that apatinib and WZB117 also inhibited the mRNA expression of PKM2. However, the synergistic inhibitory effects of the combination group were not as significant as that of the protein level **(** Figure 5H **)**. The preliminary studies suggested that IL-6 activated STAT3 and increased the expression of PKM2. We conducted a western blot test after A375, and SK-MEL-28 cells were pretreated with 20 ng/ml IL-6 for 1 h and then treated with 20 µM S31-201 (a specific STAT3 inhibitor), 20 µM apatinib combined with 22.5 µM WZB117 for 48 h, respectively. The results showed that S3I-201 (20 µM) or apatinib plus WZB117 attenuated the induction effects of IL-6 on the expressions of p-STAT3 in A375 cells. Similar to the results of A375 cells, IL-6 induced the phosphorylation of STAT3 in SK-MEL-28 cells, but S3I-201 at 20 µM concentration did not inhibit this activation. However, the combination of apatinib and WZB117 reversed the phosphorylation of STAT3 induced by IL-6 **(** Figure 5I **)**.

## Discussion

Melanoma tends to metastasize early as a highly aggressive tumor (Bastian, 2014). In recent years, targeted therapy and immunotherapy have improved the prognosis of patients with metastatic melanoma (Wolchok et al., 2022). However, in the treatment process, some patients developed drug resistance, eventually leading to disease progression and treatment failure (Welsh et al., 2016; O'Donnell et al., 2017). Thus, in the present study, we explored the new treatment strategies from the perspective of tumor metabolism. To the best of our knowledge, our study is the first to show that the combination of apatinib and WZB117 synergistically suppresses malignant phenotypes of melanoma cells.

Metabolic reprogramming is one of the hallmarks of melanoma, and melanoma cells exhibit a higher glycolytic phenotype than melanocytes (Scott et al., 2011). Unlike normal cells, the energy source of melanoma cells relies on glycolysis rather than oxidative phosphorylation (Hall et al., 2013). High expression of glycolytic regulation genes encoding glucose transporters (e.g., GLUT-1) and enzymes (e.g., PKM2) is predicted to be associated with poor survival in patients with melanoma (Falkenius et al., 2013; Koch et al., 2015; Nájera et al., 2019). Therefore, we speculated that glycolysis may be a potential target for the treatment of melanoma and studied in human melanoma cells with BRAF mutation. GLUT1 is responsible for transporting glucose into cells and plays a pivotal role in glycolysis (Fu et al., 2016). The expression of GLUT1 is upregulated in multiple solid tumors, including melanoma, and is associated with a worse prognosis (Wang et al., 2017). Inhibiting glucose uptake by small molecules has been considered a promising target for cancer therapy.

WZB117 reduces intracellular glucose levels by inhibiting GLUT1 function and has anti-tumor effects on various malignant cells (Liu et al., 2012; Li et al., 2019; Peng et al., 2019; Shima et al., 2022). Similar to previous studies, we found that WZB117 effectively inhibited the proliferation of melanoma cells. As the crystal structure of GLUT1 was revealed, glycolysis was found to be widespread in normal cells (Deng et al., 2014). GLUT1 blockade may inhibit the transporting oxygen ability of red blood cells and physiological angiogenesis, leading to side effects. Therefore, we are eager to explore other targeted drugs combined with WZB117 to reduce treatment-related toxicity at a lower dose while synergistically improving therapeutic efficacy.

Accumulating evidence has suggested that the angiogenic factors and receptors are important in regulating glycolysis and tumor progression. VEGF/VEGFR2 increases glycolysis and pathological angiogenesis in endothelial cells by upregulating the expression of GLUT1 and 6-phosphofructo-2-kinase/fructose-2,6-bisphosphatase 3 (PFKFB3) (De Bock et al., 2013; Stapor et al., 2014). Chen et al. have revealed that apatinib exerts a therapeutic effect on ovarian cancer by suppressing aerobic glycolysis via VEGFR2/AKT1 pathway inhibition (Chen et al., 2019). Besides, the persistence dependence of glycolytic metabolism in bevacizumab-resistant cells provides evidence that glycolysis inhibitors may enhance the effect of antiangiogenic therapy (Xu et al., 2013). Inhibition of glycolysis can effectively inhibit lung metastasis in a xenograft model of apatinib-resistant gastric cancer cells (Li et al., 2022). We speculate that the antitumor effect of apatinib on melanoma may be synergistic with WZB117. Our results showed that the combination of apatinib and WZB117 could synergistically inhibit cell proliferation, migration, and invasion and induce apoptosis of A375 and SK-MEL-28 cells (Figures 1D,E, 2), which is consistent with our hypothesis.

STAT3 is an oncogenic transcription factor that is constitutionally activated in various human cancers and modulates various cellular functions, such as cell proliferation, survival, apoptosis, angiogenesis, immune response, and chemotherapy resistance (Tošić and Frank, 2021). A previous study has revealed that the STAT3 pathways are one of the important downstream pathways of the VEGFR2 receptor (Bartoli et al., 2000). Multiple studies reported encouraging antitumor effects of apatinib via suppressing the VEGFR2/STAT3 pathway (Liu et al., 2017; Zheng et al., 2020). In addition, STAT3 has been found to up-regulate aerobic glycolysis and promote the proliferation of glioblastoma, bladder cancer, and breast cancer (Li et al., 2018; Cao et al., 2019; Sola-Penna et al., 2020). Previous studies have shown that STAT3 can promote glycolysis metabolism of multiple malignant tumors by regulating the expression of glycolysis key enzymes and the glycolysis regulator (Ando et al., 2010; Li et al., 2017; Hanlon et al., 2019; Darnell, 2020; Valle-Mendiola and Soto-Cruz, 2020). Our data showed that STAT3 phosphorylation level was significantly reduced in melanoma cells exposed to apatinib and WZB117 combination treatment compared to monotherapies (Figure 3 **)**. The PKM2 is one of the rate-limiting enzymes of glycolysis and is frequently upregulated in melanoma cells and tissues, promoting melanoma proliferation and progression (Falkenius et al., 2013; Nájera et al., 2019; Zhang et al., 2019). Previous studies have revealed the role of STAT3 activation in promoting glycolysis in breast cancer by upregulation of PKM2 (Sola-Penna et al., 2020). Our data suggested that silencing STAT3 can downregulate PKM2 expression while activating STAT3 can upregulate PKM2 expression in melanoma cells **(** Figure 4 **)**. Furthermore, the combination of apatinib and WZB117 inhibited both STAT3 activation and PKM2 expression (Figures 3C, 5G).

Aerobic glycolysis can produce carbon and reductive precursors to meet the needs of biosynthetic related to proliferation in various malignant tumors (Pavlova and Thompson, 2016). Although glycolysis produces less ATP than mitochondrial oxidative phosphorylation, it is more efficient in glucose utilization, produces lactic acid 10 to 100 times faster than oxidative phosphorylation, and leads to tumor microenvironment acidosis (Liberti and Locasale, 2016). Studies have shown that lactic acidosis can induce stem cell phenotype and epithelial-to-mesenchymal transition and increase cell mobility of melanoma cells *in vitro* (Andreucci et al., 2020). In addition, a xenograft model of human melanoma A375 cells pretreated under acidified conditions was established to observe accelerated tumor growth, suggesting an association between glycolysis and melanoma progression (Peppicelli et al., 2016). Our data suggested that co-treatment with apatinib and WZB117 can synergistically inhibit lactate production, glucose consumption, and PKM2 expression in A375 and SK-MEL-28 cells. Phosphorylation of STAT3 in melanoma activated by IL-6 can be reversed by co-treatment with apatinib and WZB117 **(** Figure 5 **)**. So, we speculated that the potential mechanism of malignant phenotypes suppressed by apatinib combined with WZB117 in melanoma cells might be related to the inhibition of glycolysis through the STAT3/PKM2 signaling pathway.

## Conclusion

Our study reveals that the combination of apatinib and WZB117 displays synergistic anti-tumor activity against melanoma via blocking STAT3/PKM2 axis and is a potential therapeutic strategy for melanoma.

## Data Availability

The original contributions presented in the study are included in the article/supplementary material, further inquiries can be directed to the corresponding author.
